# Supplementary material for: Structural mechanism of laminin recognition by integrin
Source: Nat Commun. 2021 Jun 29;12:4012. doi: 10.1038/s41467-021-24184-8 (PMC8241838; doi:10.1038/s41467-021-24184-8)
Supplement: Supplementary file 1 — Supplementary Figures and Tables [file 41467_2021_24184_MOESM1_ESM.pdf]

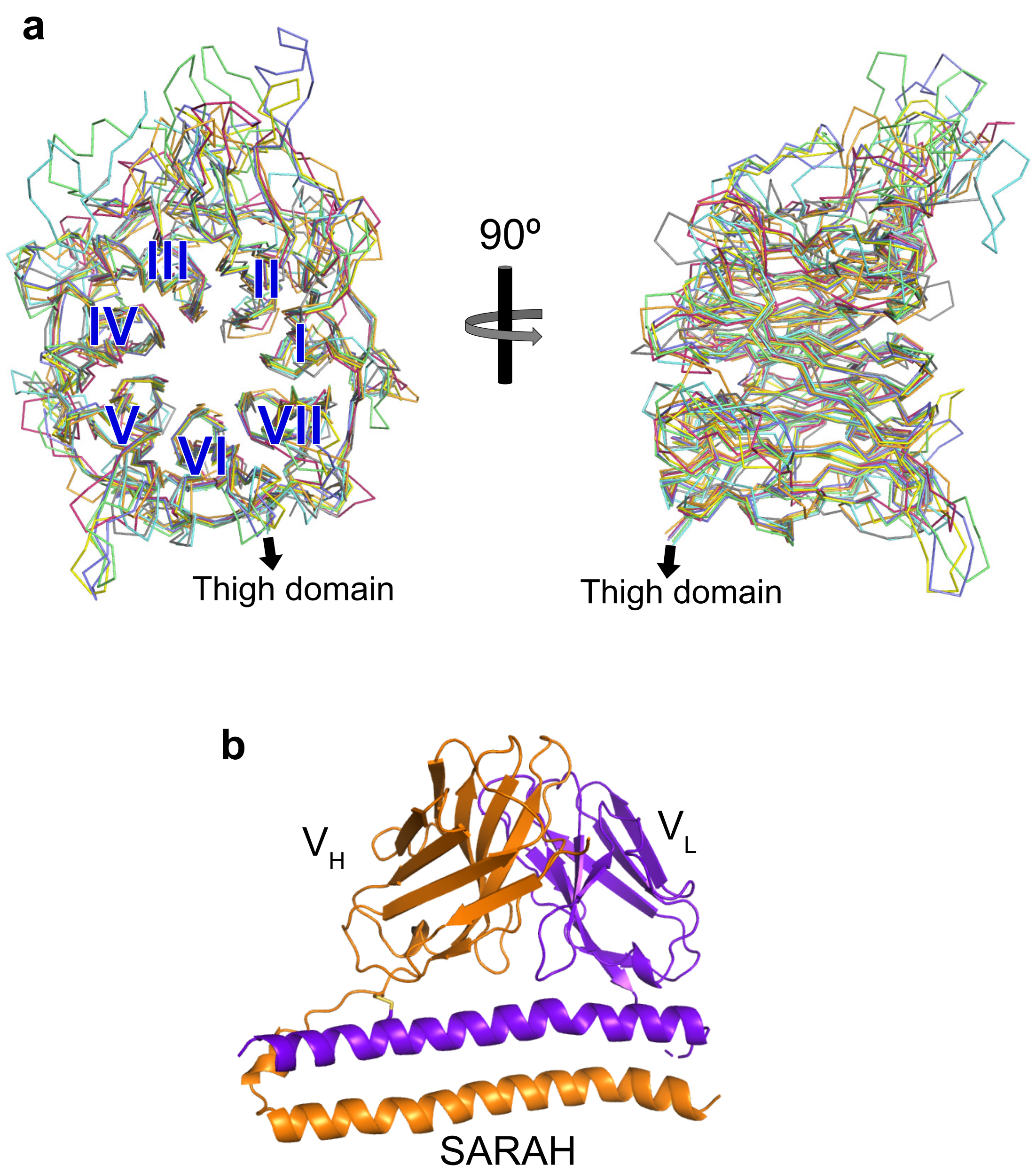

Supplementary Fig. 1 Evaluation of the crystal structures.
a, Structural comparison of the β-propeller domains in the available α subunit structures. The β-propeller domain structures of α6 (magenta), α5 (PDB ID: 4wk0, slate), αV (PDB ID: 5ffg, yellow), αIIb (PDB ID: 3fcs, green), α4 (PDB ID: 4irz, orange), αL (PDB ID: 5e6s, gray), and αX (PDB ID: 4neh, cyan) are superposed and shown as Cα-trace representations. The 7 β-sheets are denoted as blades I through VII and labeled. **b**, Crystal structure of the HUTS-4 Fv-clasp determined prior to the model building of the quaternary complex. V_H-SARAH and V_L-SARAH are shown in orange and purple, respectively.

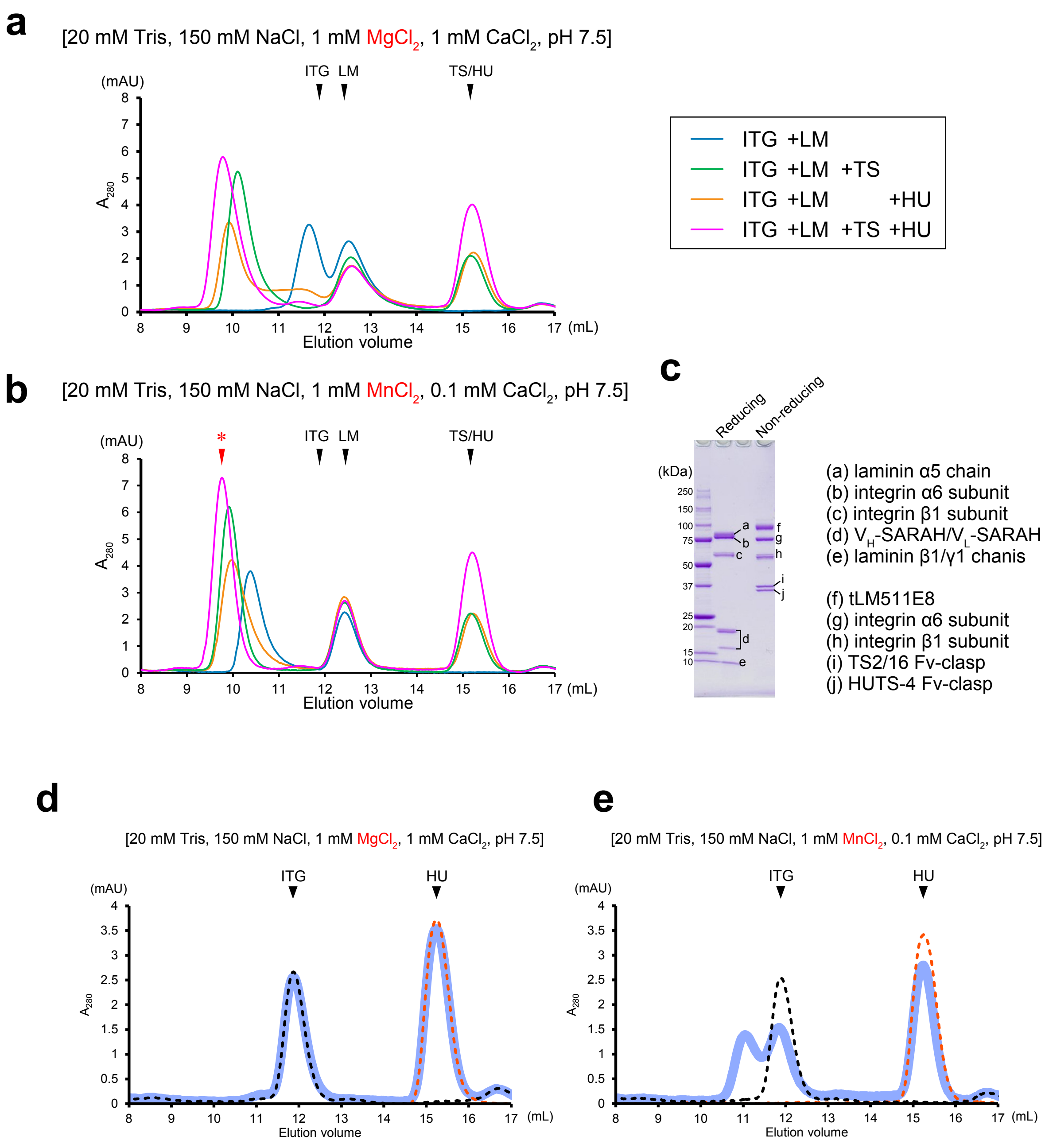

Supplementary Fig. 2 Complex formation analyzed by size-exclusion chromatography (SEC). **a, b,** SEC profiles of the mixtures of samples in the presence of 1 mM $MgCl_2$/1 mM $CaCl_2$ (**a**) or 1 mM $MnCl_2$/0.1 mM $CaCl_2$ (**b**). Chromatograms with different sample composition are shown in differently colored lines indicated in the key. ITG, α6β1 headpiece; LM, tLM511; TS, TS2/16 Fv-clasp, and HU, HUTS-4 Fv-clasp. The elution positions for individual component alone are indicated by black arrowheads. **c**, SDS-PAGE profile of the peak fraction sample (marked by a red asterisk in (b)) used for the cryo-EM observation. This experiment was repeated twice. Un-cropped gel image is provided in the Source Data file. **d, e,** Binding of HUTS-4 Fv-clasp to α6β1. SEC profiles in the presence of 1 mM $MgCl_2$/1 mM $CaCl_2$ (**d**) or 1 mM $MnCl_2$/0.1 mM $CaCl_2$ (**e**) are shown. The buffer compositions are provided above each chromatogram. SEC profiles of integrin alone (black dashed line), HUTS-4 Fv-clasp alone (orange dashed line), and their 1:1.5 mixture (thick blue line) are shown. The elution positionsfor individual component alone are indicated by black arrowheads (ITG, α6β1 headpiece; HU, HUTS-4 Fv-clasp).

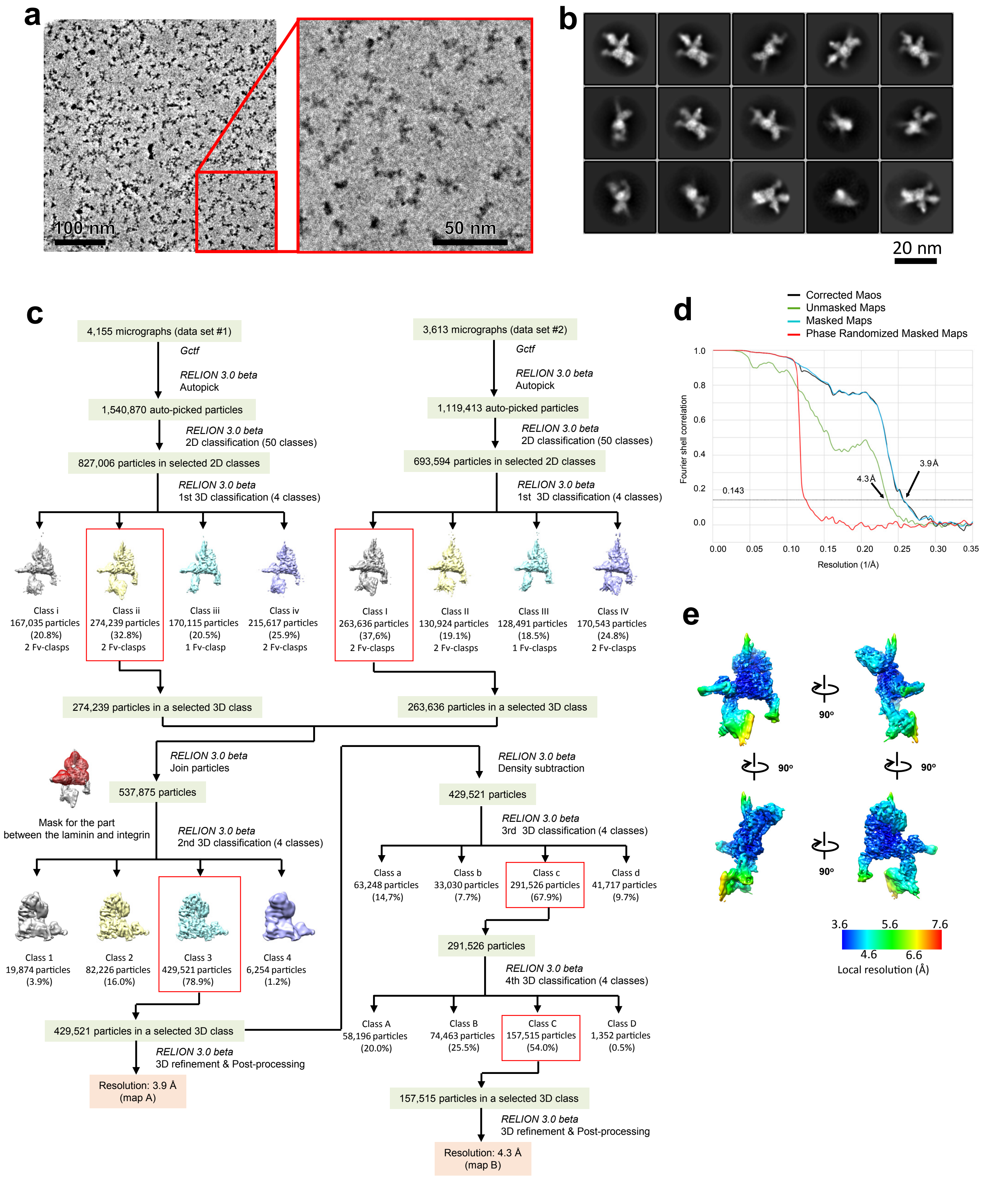

Supplementary Fig. 3 Single-particle analysis of the α6β1 headpiece-tLM511 complex. **a**, Representative cryo-EM micrograph of the α6β1-tLM511 complex arbitrary chosen by visual inspection of field views from >4,000 collected images. **b,** Representative 2D classes of the α6β1-tLM511 complex. **c**, A schematic flowchart of the cryo-EM data collection, classification, and refinement. For the detail, see the Method section. **d**, The gold standard FSC curve. **e**, The local resolution map of the complex viewed from four different orientations and colored based on the local resolution scale shown in the bottom.

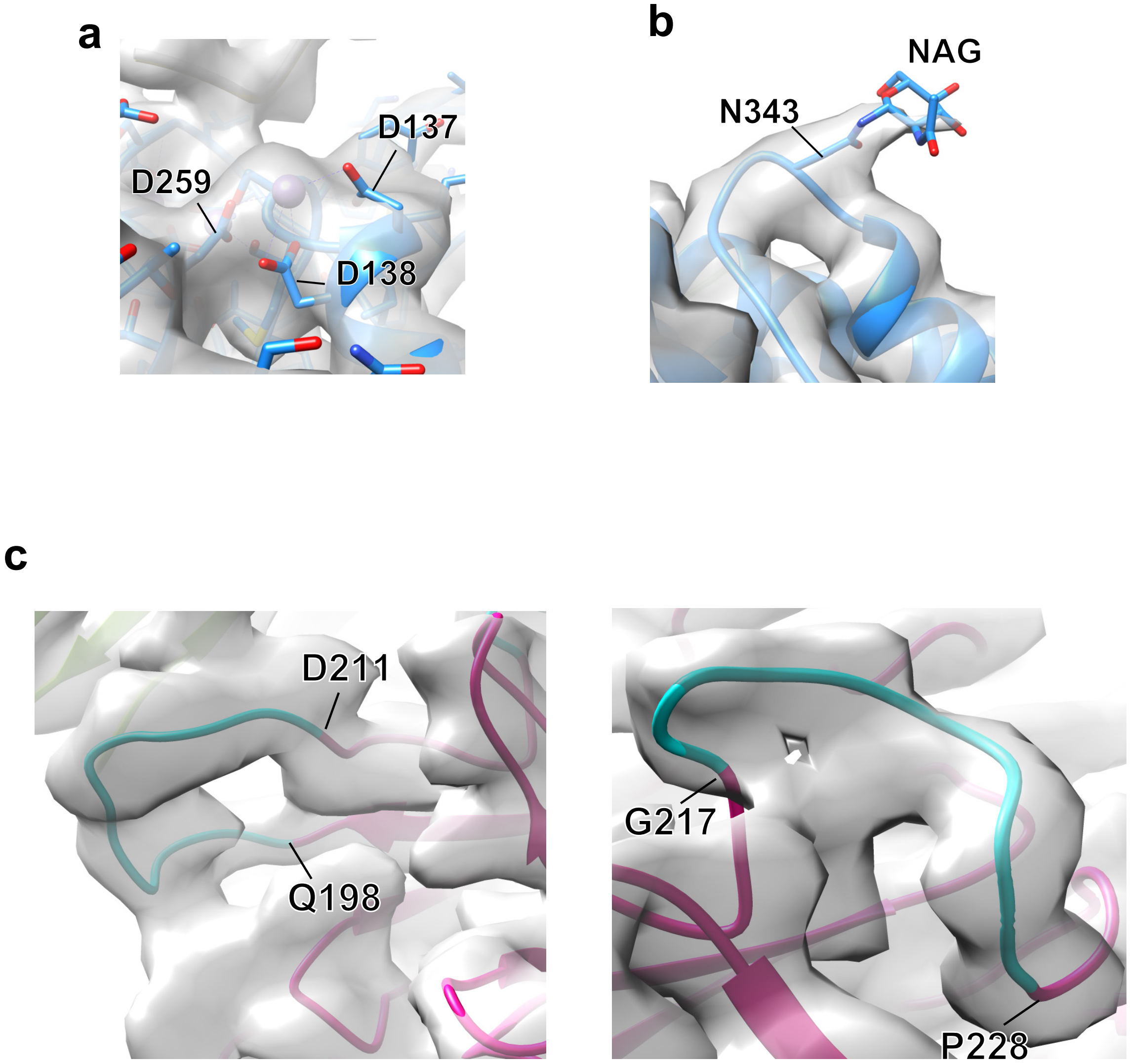

Supplementary Fig. 4 Cryo-EM maps.
a, Cryo-EM map near the ADMIDAS. The presence of a metal at this site is inferred by the observed volume surrounded by ADMIDAS-coordinating D137, D138, and D259. **b**, Cryo-EM map of the α6β1-tLM511 complex near the *N*-acetylglucosamine (NAG)-bearing N343 located in the β6-α7 loop of the βI domain. **c**, Cryo-EM map for the X1 region of integrin α6 in the complex. Putative Cα path for the segments not included in the final model are shown in a different color (cyan) than the modeled regions (magenta). All maps are drawn at the contour level of 0.05 in Chimera.

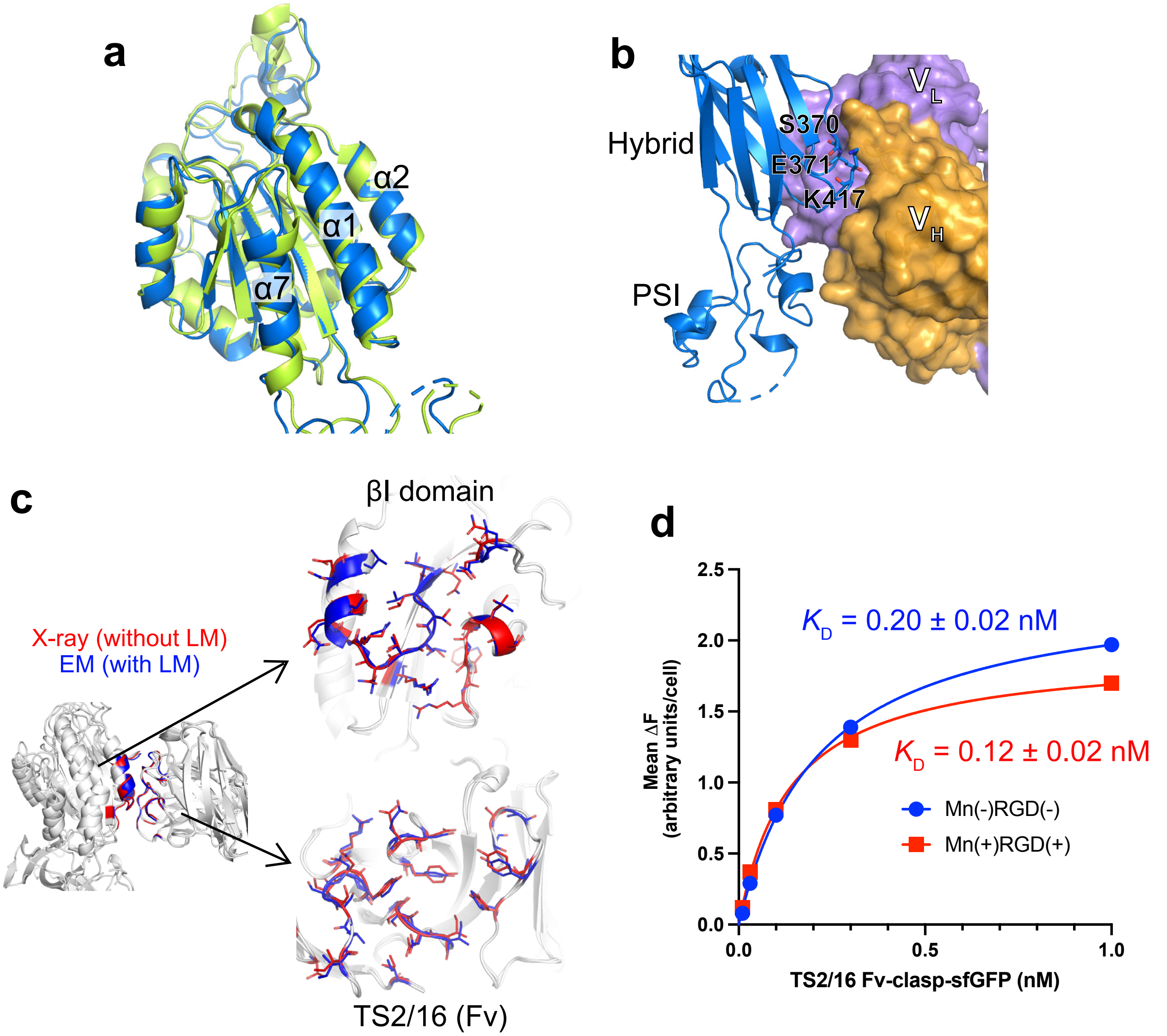

Supplementary Fig. 5 Open conformation of the integrin β1 chain and its interface with anti-β1 antibodies.

a, Structural comparison of the βI domain in the α6β1-tLM511 complex (sky blue) with that of αIIbβ3 in the open-head conformation (PDB ID: 2vdo, light green). The two structures are superposed well, including the conformations of α1 and α7 helices. **b**, HUTS-4 binding site. HUTS-4 (surface model) recognizes continuous surface of β1 upper leg made by the hybrid domain and the upper portion of the PSI domain (ribbon model). Species-specific epitope residues determined previously (i.e., S370, E371, K417) are shown as stick models. **c**, Structural comparison of TS2/16 binding interface between the ligand unbound and bound states. Residues making a direct contacts (< 4Å) are highlighted in red (ligand unboundcrystal structure) or blue (ligand-bound cryo-EM structure) and shown as stick models. The average Cα displacements upon the ligand binding are 0.468 Å and 0.509 Å for 16 βI domainresidues and 17 TS2/16 residues, respectively. **d**, Affinity measurement of monomeric TS2/16 toward α5β1 on K562 cells. Concentration-dependent binding of sfGFP-fused TS2/16 Fv-clasp was evaluated as described in the Method under the resting (in the presence of 1 mM $CaCl_2$ and 1 mM $MgCl_2$, blue) or maximally activated (in the presence of 0.5 mM $MnCl_2$, and 1 mM RGD peptide, red) conditions, and the equilibrium dissociation constants (K_D) were derived from the binding isotherms by non-linear regression analysis. Data are mean ± SD of three independent experiments, while the plots are from one representative experiment among them. Source data are provided as a Source Data file.

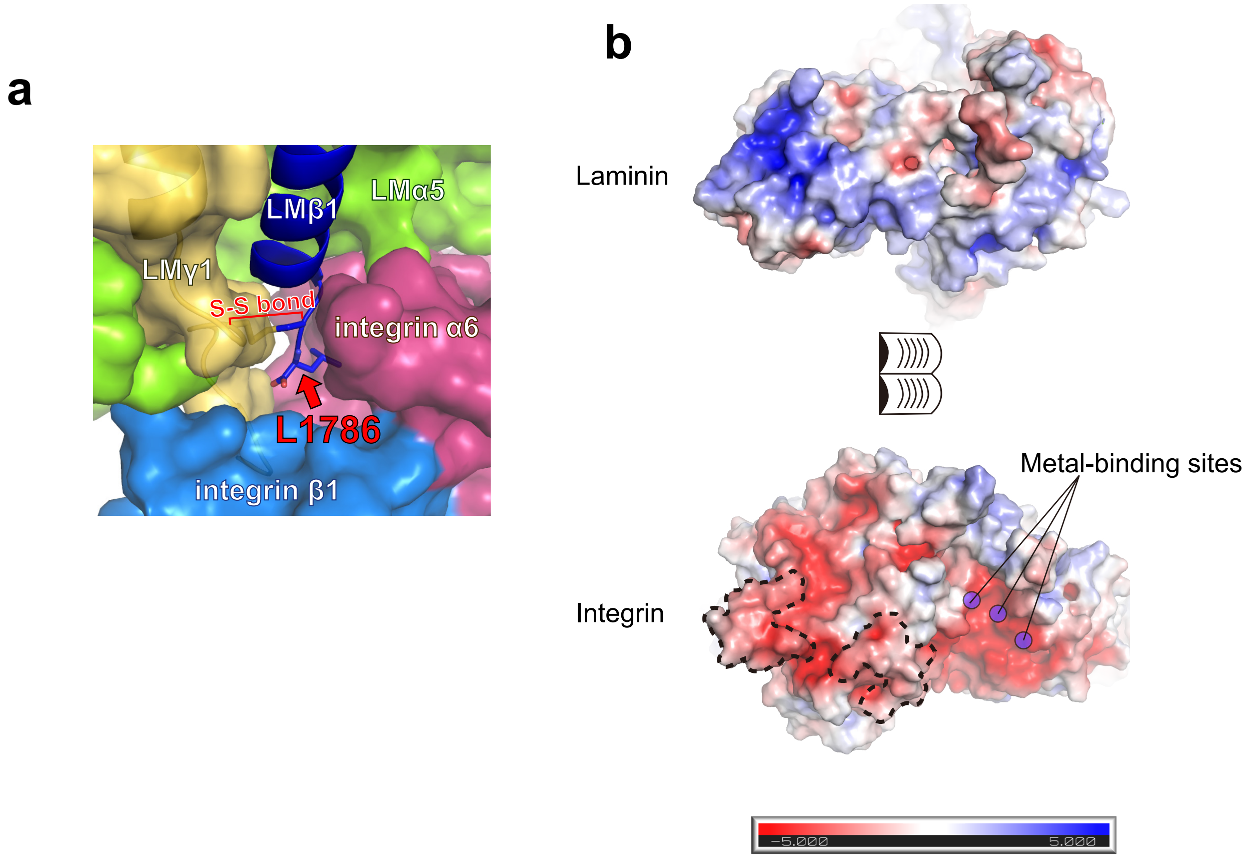

Supplementary Fig. 6 Closer look at the α6β1-LM511 interface.
a, The C-terminus of the LMβ1 chain contributes to the binding interface. The C-terminal L1786 (stick model) of the laminin β1 chain (dark blue) is accommodated in a small dead-end space formed by integrin α6 (hot pink) and β1 (sky blue) subunits and the laminin γ1-tail (yellow). **b**, Electrostatic complementarity of the α6β1-LM511 interface. Electrostatic surfaces of LM511 (top) and α6β1 (bottom) with a contour level of ±5 kT/e are shown as an open book view similar to Fig.5a, except for using the α6 coordinates complemented with the putative models for loops flanking strand IIId (corresponding regions indicated by dotted lines). Note that β1 surface near the three metal-binding sites (blue dots) appearing strongly electronegative is partially neutralized by the positive charge of the metals.

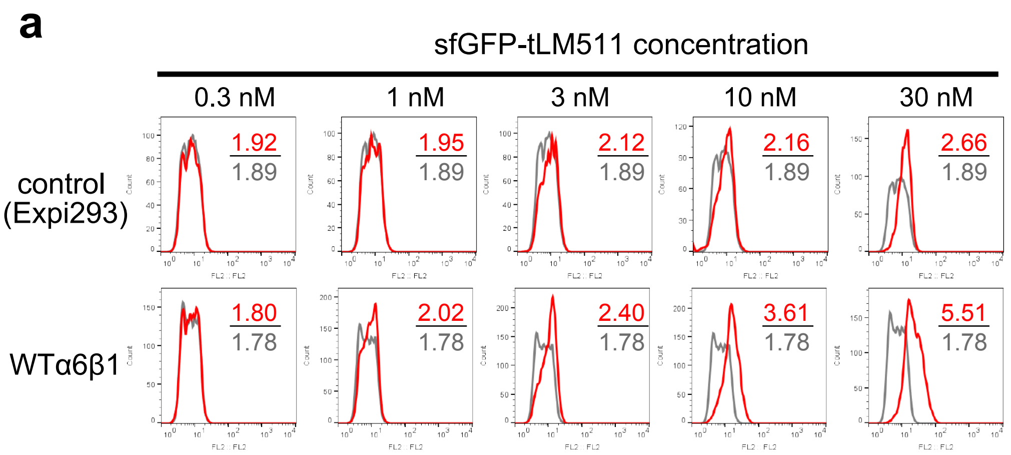

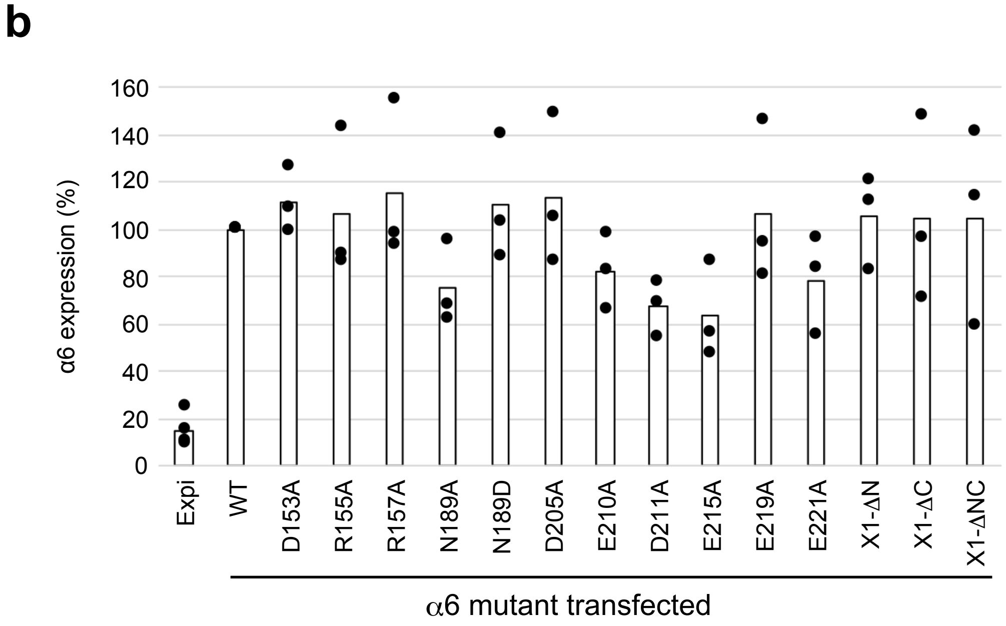

Supplementary Fig. 7 Binding of sfGFP-tLM511 to cell-surface α6β1 integrin measured by flow cytometry.

a, Concentration-dependent binding of sfGFP-tLM511. Either control Expi293F cells or the same cells transfected with wild-type α6 and β1 integrin subunits were incubated with purified sfGFP-tLM511 for 2h in the presence of 0.5 mM $MnCl_2$, and directly analyzed in a flow cytometer without any washing steps. The fluorescence signal associated with each cell is extracted from the acquired data in a linear scale, and values from all gated cells were integrated to obtain mean cellular fluorescence in an arbitrary unit, shown in red. These numbers, after subtracting the value from the histogram obtained with no sfGFP-tLM511 (gray histogram and gray numbers), were used to plot binding isotherms shown in Fig. 6d-f. **b**, Expression levels of full-length integrins. Cell-surface expression levels for various mutant α6 subunits were evaluated by measuring the mean fluorescent intensity (MFI) from the FACS staining using an anti-α6 integrin antibody GoH3 (#MAB1378, Merck-Millipore). The MFI values, after subtracting the value obtained with the control sample without GoH3, are normalized to that of wild-type (WT) and expressed as %. Shown are mean value of three independent measurements (rectangles), with each data point overlaied as black dot. Note that untransfected Expi293F cells show presence of endogenous α6 subunit at a level ~15% of the WT α6 overexpression. Source data are provided as a Source Data file.

a

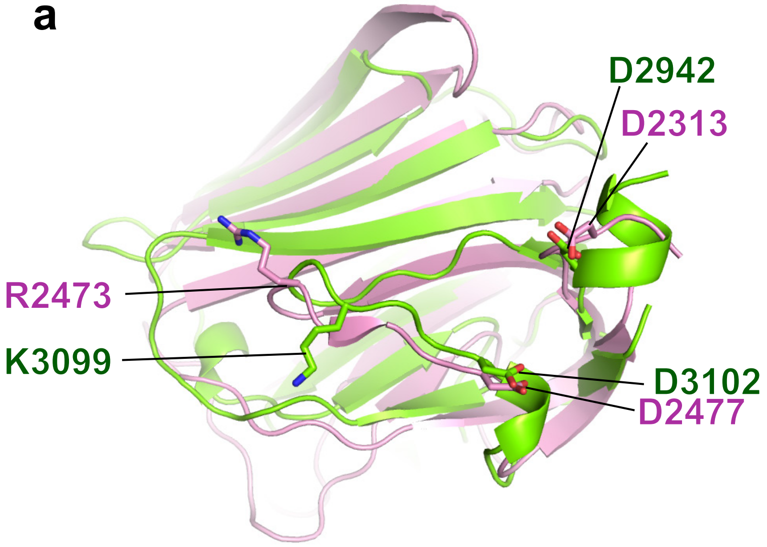

b

```
             2942
hLMα5 :   DPWLTDG-SYLDGTGFARISFDSQI-STTKRFEQELRLVSYSGVLFFLKQ--QSQFLCLAVQEGSLVLLYDFGAG
mLMα1 :   ---NEDSSFHFDGSGYAMVEKTLR--PTVTQIVILFSTFSPNGLLFYLASNGTKDFLSIELVRGRVKVMVDLGSG
hLMα1 :   ---NEDPSFHFDGSGYSVVEKSLP--ATVTQIIMLFNTFSPNGLLLYLGSYGTKDFLSIELFRGRVKVMTDLGSG

hLMα5 :   LKKAVPLQPPPPLTSASKAIQVFLLGGSRKRVLVRVER-----ATVYSVE------QDNDLELADAYYLGGVPPD
mLMα1 :   PLTLMT--DRRYNNGTWYKIAFQRN--RKQGLLAVFDAYDTSDKETKQGETPGAASDLNRLEKDLIYV-GGLPHS
hLMα1 :   PITLLT--DRRYNNGTWYKIAFQRN--RKQGVLAVIDAYNTSNKETKQGETPGASSDLNRLDKDPIYV-GGLPRS

                                      3099 3102
hLMα5 :   QLPPSLRRLFPTGGSVRGCVKGIKALGKY-VDLKRLNTTGVSAG
mLMα1 :   KAV----RKGVSSRSYVGCIKNL-EISRSTFDLLRNS-YGVRKG
hLMα1 :   RVV----RRGVTTKSFVGCIKNL-EISRSTFDLLRNS-YGVRKG
```

Supplementary Fig. 8 Comparison of the laminin LG2 domain.
a, Superposition of the LG2 domains of LM511 (cryo-EM structure, light green) and mLM111 (PDB ID: 5mc9, pink). The charged residues on LM511 where mutations were introduced in this study and their corresponding residues on mLM111 are shown as stick models. **b**, Sequence alignment of the LG2 domains among human laminin α5, mouse laminin α1, and human α1.

Supplementary Table 1 X-ray diffraction data collection and refinement statistics

	α6β1 headpiece-TS2/16 Fv-clasp complex (PDB ID: 7ceb)	HUTS4 Fv-clasp (PDB ID: 7cea)
**Data collection**		
Space group	$P2_12_12$	$P3_221$
Cell dimensions		
a, *b*, *c* (Å)	116.5, 251.3, 63.3	88.4, 88.4, 78.1
Resolution (Å)	47.83 – 2.89 (3.07 - 2.89)[a]	44.18 - 2.55 (2.70 - 2.55)
R_{sym}	0.16 (1.23)	0.10 (2.16)
$I / \sigma I$	12.48 (1.63)	17.75 (1.75)
CC1/2	0.997 (0.806)	0.999 (0.899)
Completeness (%)	99.6 (97.7)	99.8 (99.2)
Redundancy	9.8 (9.8)	13.5 (14.2)
**Refinement**		
Resolution (Å)	44.6 - 2.89 (2.96- 2.89)	34.79 - 2.55 (2.8 - 2.55)
No. reflections	42,218	11,755
R_{work} / R_{free} (%)	20.7/24.9 (33.1/39.0)	25.2/26.6 (42.5/43.2)
No. atoms		
Proteins	9,540	2,565
Carbohydrates	84	-
Metal ions	5	-
Water	3	-
B-factors		
Proteins	78.5	92.3
Carbohydrates	97.9	-
Metal ions	83.2	-
Water	62.8	-
R.m.s. deviations		
Bond lengths (Å)	0.004	0.005
Bond angles (°)	0.774	0.867
Validation		
MolProbity score	1.74	2.00
Clashscore	8.45	9.06
Poor rotamers (%)	0	1.76
Ramachandran plot		
Favored (%)	95.90	95.31
Allowed (%)	4.10	4.69
Disallowed (%)	0	0
Cβ outliers (%)	0	0

A single crystal was used for each structure determination.
[a]Values in parentheses are statistics of the highest-resolution shell.

Supplementary Table 2 Cryo-EM data collection, refinement and validation statistics

	α6β1 headpiece-tLM511 complex (EMDB-30342) (PDB 7cec)
**Data collection and processing**	
Magnification	59,000
Voltage (kV)	300
Electron exposure (e–/Å^2)	40
Defocus range (μm)	−0.6 ～ −0.8
Pixel size (Å)	1.113
Symmetry imposed	C1
Initial particle images (no.)	2,660,283
Final particle images (no.)	429,521
Map resolution (Å)	
FSC threshold	0.143
Map resolution (Å)	3.9
**Refinement**	
Initial model used (PDB code)	7ceb, 4wk0, 5xau, 5xcx, 7cea
Model resolution (Å)	3.9
FSC threshold	0.5
Map sharpening *B* factor (Å^2)	-100
Model composition	
Non-hydrogen atoms	17,561
Protein residues	17,442
Carbohydrates	112
Metal ions	7
B factors (Å^2)	
Protein	172.1
Carbohydrates	172.0
Metal ions	152.3
R.m.s. deviations	
Bond lengths (Å)	0.011
Bond angles (°)	1.202
Validation	
MolProbity score	2.22
Clashscore	14.95
Poor rotamers (%)	1.09
Ramachandran plot	
Favored (%)	91.26
Allowed (%)	8.69
Disallowed (%)	0.05

Supplementary Note

Formation of a stable complex of α6β1 integrin with LM511

We explored conditions where a stable complex of α6β1 with laminin is obtained, a prerequisite for the structural determination of the complex. Previously, we have established that a recombinantly produced truncated E8 fragment of LM511 (tLM511) retained full integrin-binding activity as well as an excellent crystallization property [12]. Thus we evaluated the stability of the complex between the α6β1 headpiece and tLM511 by using a size-exclusion chromatography (SEC). When the mixture was subjected to SEC in a buffer containing physiological concentration of divalent cations (*i.e.*, 1 mM $MgCl_2$/1 mM $CaCl_2$), both components eluted as uncomplexed species (Supplementary Fig. 2a, blue tracing) indicating a fast dissociation rate of the complex. In contrast, addition of TS2/16 resulted in the formation of stable complex appearing as a peak at ~10.1 ml (Supplementary Fig. 2a, green), although some dissociation was suspected by the trailing shape of the peak. Furthermore, the peak shape became nearly symmetrical when 1 mM Mn^{2+} was included in the elution buffer (Supplementary Fig. 2b, green), suggesting that the most stable complex can be obtained in this condition. When the complex sample thus prepared was subjected to extensive crystallization screening, however, we could not obtain any diffraction-compatible crystals. We went further to include yet another anti-integrin antibody that may reduce the conformational heterogeneity. HUTS-4 is an anti-integrin β1 antibody whose epitope is located in β1 hybrid domain, which is predicted to be inaccessible when β1 assumes low-affinity closed conformation but exposed when it is in the activated, open conformation [40]. This makes it one of the special anti-integrin antibodies collectively called ligand-induced binding site (LIBS) antibodies, and we speculated that HUTS-4 may stabilize the open-leg conformation of α6β1 headpiece and facilitate the crystallization. We prepared HUTS-4 Fv-clasp and tested its binding to α6β1 by SEC under various conditions. It was revealed that HUTS-4 failed to bind in a physiological buffer (*i.e.*, 1 mM Mg^{2+} and Ca^{2+})(Supplementary Fig. 2d), and bound only partially in the presence of 1 mM Mn^{2+} (Supplementary Fig. 2e). Although inclusion of the ligand (*i.e.*, tLM511) induced HUTS-4 binding in both conditions (Supplementary Fig. 2a, b, orange tracings), the shape of the elution peaks suggested incomplete formation of the complex, particularly under physiological cation conditions. Finally, we mixed all the components (α6β1 headpiece, tLM511, TS2/16 Fv-clasp, and HUTS-4 Fv-clasp) in the presence of 1 mM Mn^{2+}, which gave very sharp peak in SEC (Supplementary Fig. 2b, magenta tracing) containing all components as confirmed by SDS-PAGE analysis (Supplementary Fig. 2c). This preparation was subjected to the cryo-EM single particle analysis and we succeeded in obtaining a 3.9Å-resolution structure of the quaternary complex.
