# Supplementary material for: Structural mechanism of laminin recognition by integrin
Source: Nat Commun. 2021 Jun 29;12:4012. doi: 10.1038/s41467-021-24184-8 (PMC8241838; doi:10.1038/s41467-021-24184-8)
Supplement: Supplementary file 4 — Description of additional supplementary files [file 41467_2021_24184_MOESM4_ESM.docx]

Description of additional supplementary information files

Title: Structure of a quaternary complex of the α6β1 headpiece, tLM511, TS2/16, and HUTS-4

Description: Entire view of cryo-EM map of the quaternary complex rotated around the vertical axis, with the structural model (in cartoon presentation) embedded in the map. Each domain is colored as in the figure 3.
